# Rationale and design of the application value of Beijing Vascular Health Stratification (BVHS): predictive value of combined assessment of vascular structure and function for cardiovascular events in general Chinese population

**DOI:** 10.1186/s12872-021-02289-8

**Published:** 2021-10-15

**Authors:** Huan Liu, Xiaohua Zhou, Jinbo Liu, Wei Huang, Na Zhao, Hongyu Wang

**Affiliations:** 1 Vascular Medicine Center, Peking University Shougang Hospital, No. 9 Jinyuanzhuang Road, Shijingshan District, Beijing, 100144 People’s Republic of China; 2 Vascular Health Research Center of Peking University Health Science Center, No. 9 Jinyuanzhuang Road, Shijingshan District, Beijing, 100144 People’s Republic of China; 3 Key Laboratory of Molecular Cardiovascular Sciences (Peking University), Ministry of Education, No. 38 Xueyuan Road, Haidian District, Beijing, 100191 People’s Republic of China; 4 Peking University Clinical Research Institute, Peking University Health Science Center, No. 38 Xueyuan Road, Haidian District, Beijing, 100191 People’s Republic of China; 5 Department of Biostatistics and Beijing International Center for Mathematical Research, Peking University, No. 5 Yiheyuan Road, Haidian District, Beijing, 100871 People’s Republic of China

**Keywords:** Beijing Vascular Health Stratification, Vascular health, Cardiovascular disease, Risk assessment, Prediction

## Abstract

**Background:**

Vascular endothelial dysfunction, arteriosclerosis and atherosclerotic plaque are well-known risk factors for cardiovascular disease (CVD). Studies on vascular health markers have been well-established, however, there is still a lack of related research on combined vascular structure and function indicators.

**Method:**

Beijing vascular health stratification (BVHS) is an evaluation system aiming at vascular health, combined the endothelial function, arteriosclerosis, atherosclerotic plaque and vascular lumen stenosis to comprehensively assess the vascular health and grade it. This study will explore the predictive value of the combined evaluation of vascular structure and function for cardiovascular events and assess the predictive value of BVHS and compare it with the existing risk assessment systems. A total of 1500 subjects will be enrolled into the prospective cohort study from a community and will be followed up for at least 3 years from July 1, 2020 to June 30, 2023. Subjects aged 40 or above, without coronary heart disease, stroke or peripheral artery disease, with written informed consent will be included; subjects with end-stage hepatorenal diseases (uremia, renal failure, cirrhosis, liver failure), mental disorders or cognitive disorders, with any other factors that the researcher thinks are not suitable for the study will be excluded. Traditional cardiovascular risk factors will be collected as adjusted confounders.

**Discussion:**

BVHS is a potential and scientific vascular health evaluation system. The study will be the first to grade vascular health by combing various vascular indicators and explore the prediction value and compare with other risk prediction system in general Chinese population.

*Trial registration*: The trial is registered on http://www.chictr.org.cn/ (ChiCTR2000034085).

## Background

Vascular health indicators, including lumen stenosis and dysfunction, both reflect the long-term cumulative effects of traditional and unidentified cardiovascular (CV) risk factors before and after clinical vascular events, and can be regarded as an alternative end point indexes for target organ damage and risk prediction [1]. Several markers have been considered as the reflection of vascular health, among them endothelial function, arterial stiffness and carotid atherosclerosis are the most common indicators [1]. Endothelial dysfunction may represent the effect of traditional CV risk factors on vascular health [2]. Arterial stiffness is increasingly recognized as a surrogate end point for cardiovascular disease (CVD) and as a risk factor for clinical hypertension [3, 4]. Carotid ultrasound measurement of intima-media thickness (IMT) along with carotid plaque are emerging as the focus of carotid artery ultrasound imaging for CV risk prediction [5]. Currently, studies on assessing vascular health use only one or more of these indicators [6–8]. Beijing Vascular Health Stratification (BVHS) was put forward to assess vascular health including all stages of vascular disease progression, from endothelial function to arteriosclerosis to vascular stenosis [9]. BVHS is a risk prediction tool using subclinical vascular measures such as endothelial function, arterial stiffness, carotid atherosclerotic plaques and artery stenosis by non-invasive detections. Furthermore, traditional risk factors are not included in BVHS, but are used as adjusted confounding factors to analyze the independent predictive role of BVHS.

All the markers are graded as artery functional injury or structural disease or both. Our previous retrospective study found that the BVHS was a comprehensive risk assessment system and was independent of traditional CV risk factors [10]. However, the previous study did not develop a strict study design and follow-up plan. Therefore, this study was designed as a prospective cohort study to verify the previous research results and further explore the clinical value of BVHS for the prediction of major adverse cardiovascular events (MACEs).

## Aim

BVHS Study is designed to answer two important clinical questions: (1) A preliminary study on the value of BVHS in predicting MACEs independently of traditional CVD risk factors; and (2) A preliminary comparative study on the predictive value of BVHS and other risk prediction system model of CVD commonly used at home and abroad, such as the China-PAR Project [11] and the Pooled Cohort Equations [12].

## Methods

### Study design (Fig. 1)

**Fig. 1:**
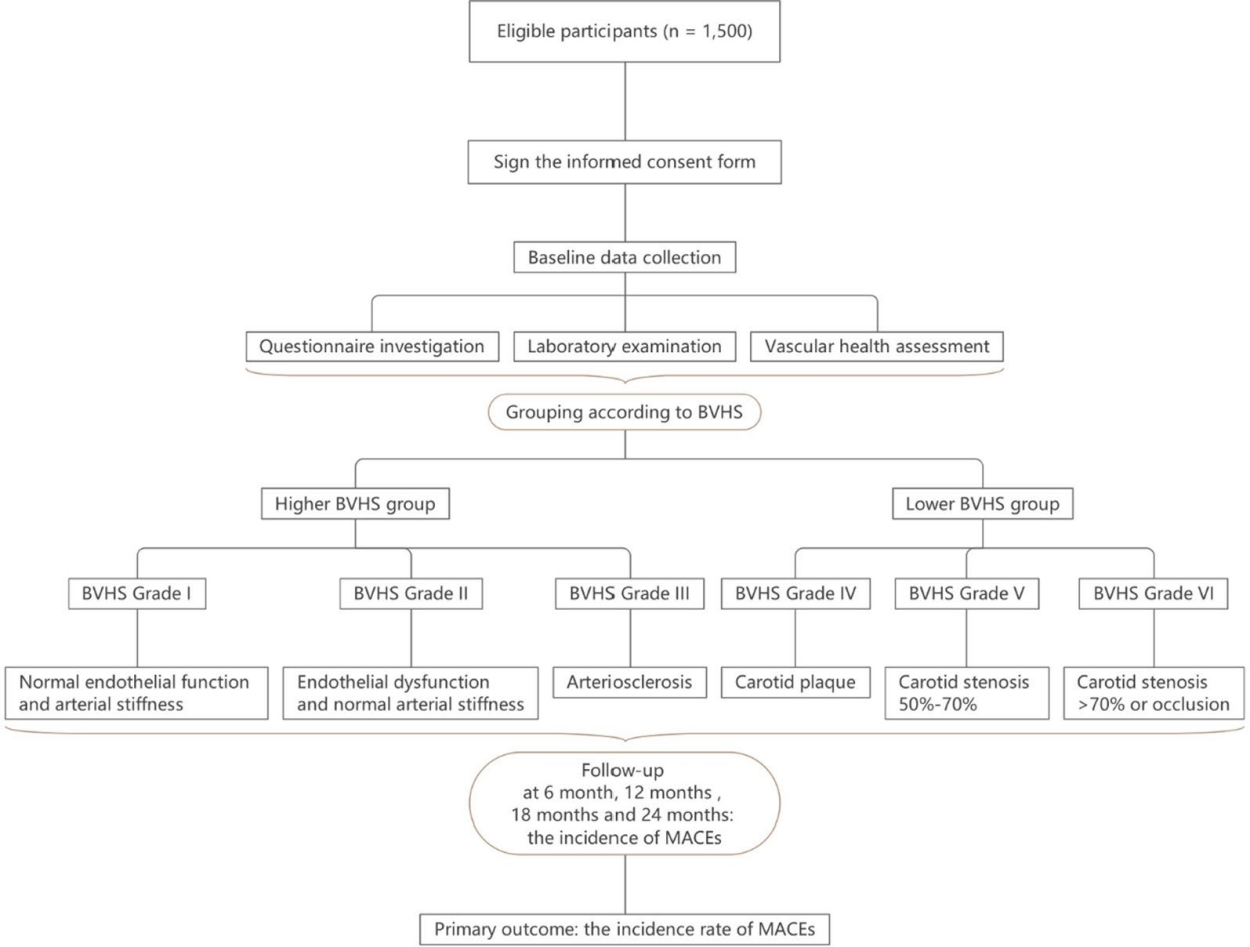
Study design. BVHS, Beijing Vascular Health Stratification; MACEs, major adverse cardiovascular events

BVHS is a prospective cohort study which will enroll 1500 participants from Jindingjie community health service center in western Beijing, China. The biggest advantage of the community population is that the participants are permanent residents of the area, have good compliance, and are easy to carry out follow-up studies.

The study design is showed in Fig. 1.

### Participants population, inclusion and exclusion criteria

Participants without CVD from Jindingjie community health service centers will be recruited by their site-specific responsible physicians.

Subjects aged 40 or above, without coronary heart disease, stroke or peripheral artery disease, with written informed consent will be included; subjects with liver disease (hepatic jaundice, cirrhosis or liver failure), chronic kidney disease (estimated Glomerular Filtration Rate, eGFR < 60 mL/min per 1.73 m^2^), mental disorders or cognitive disorders, with any other factors that the researcher thinks are not suitable for the study such as non-local residents or life expectancy less than one year will be excluded.

### Baseline demographics, physical examinations and data collection

Case record form (CRF) and electronic data capture (EDC) are used for data collection and data management. The personal information of all participants will be deprivileged.

Data collected at the baseline will include participants’ demographics including date of birth, gender, education, occupation, and health insurance, lifestyles including tobacco smoking, alcohol drinking, diet pattern (food frequency), and time on sedentary life, systolic blood pressure (SBP), diastolic blood pressure (DBP), heart rate, height, weight; medical history of hypertension, diabetes mellitus and hyperlipidemia, and current use of medications (statins, aspirin/clopidogrel, angiotensin converting enzyme inhibitors/angiotensin receptor blocker, β-receptor blocker, calcium channel blockers, insulin, hypoglycemic medicine, and nitrates). Blood pressure and heart rate were measured by pulse wave sphygmomanometer (RBP-9000c, Shenzhen, China) for 3 times after 30 s interval. Baseline laboratory blood parameters to be collected include total cholesterol (TC), triglyceride (TG), low density lipoprotein cholesterol (LDL-C), high density lipoprotein cholesterol (HDL-C), C-reactive protein (CRP), homocysteine (HCY), uric acid, urea nitrogen, creatinine, fasting plasma glucose (FPG), alanine aminotransferase (ALT), aspartate aminotransferase (AST), total protein, albumin, lactate dehydrogenase, hydroxybutyric acid, creatine kinase, creatine kinase isoenzyme.

Hypertension is defined as SBP ≥ 140 mmHg or DBP ≥ 90 mmHg or current use of anti-hypertension medications. Diabetes mellitus is defined as either glycosylated hemoglobin (HbA1c) ≥ 6.5% or FPG ≥ 126 mg/ dL (7.0 mmol/L) or 2-h plasma glucose ≥ 200 mg/dL (11.1 mmol/L) during an oral glucose tolerance test; or currently taking blood glucose lowering medications or insulin. Hyperlipidemia includes the following: TC > 200 mg/dL/(5.18 mmol/L); or LDL-C ≥ 130 mg/dL (3.37 mmol/L); or HDL-C < 40 mg/dL (1.04 mmol/L) in men and < 50 mg/dL (1.30 mmol/L) in women; or lipoprotein a > 50 mg/dL (125 nmol/L), or persistent elevations of TG ≥ 175 mg/dL (≥ 1.97 mmol/L); or currently receiving antilipidemic medications.

### Vascular health evaluation

#### Endothelial function by peripheral arterial tonometry (PAT)

Non-invasive peripheral endothelial function will be assessed by reactive hyperemia index (RHI) using Endo PAT 2000 Machine (Itamar Medical Ltd, Caesarea, Israel). Endothelial function test will be administrated by a trained staff in a separate room and performed in the morning or early afternoon (starting time between 7:30 AM and 11:00 AM). The Endo-PAT data will be analyzed with the proprietary software package, without any input from the examiner and has been developed to measure observer independent pulsatile arterial volume changes by finger plethysmography. The temperature of the testing environment is 22–25 °C, dim the lights and keep quiet. The required equipment includes a comfortable examination bed, a computer, Endo-PAT equipment, hand support, and a blood flow occlusion meter. The test takes a total of 16 min, including baseline 6 min, interruption of 5 min, and release of 5 min. The subject takes a supine position, relax, and his arms can be placed flat on both sides of the body. Subjects avoid smoking, eating food, coffee or other drinks (drinking water is allowed) at least 3 h before the test. The biosensor is worn on the index finger (second finger) and bind the cuff of the non-habitual hand to block the blood flow. Tentatively, the RHI < 1.67 is regarded as endothelial dysfunction. At present, there is no study on the normal reference value of RHI, and our study will also preliminarily establish the normal range of RHI in Chinese population.

#### Arterial stiffness by brachial-ankle artery pulse wave velocity (ba-PWV) examination

Ba-PWV is examined by automatic detection equipment for arterial function (MB-3000, China). Keep the room temperature at about 22–25 °C in the examination room. The examinee should rest for at least 5 min before the measurement; rest for about 20 min after any exercise before starting the measurement. Install cuff on both arms and ankles, left upper arm (yellow), right upper arm (red), left ankle (green), right ankle (black). The air duct orifice of the upper arm cuff is placed on the same axis as the brachial artery of the upper arm, and the lower edge of the cuff is 2 to 3 cm, from the elbow fossa so that the upper and lower edges can only enter one finger. Extend the air hose on the ankle cuff upward along the medial side of the ankle. The lower edge of the lower limb cuff is 1 to 2 cm horizontally away from the medial malleolus. Place electrocardiogram (ECG) electrodes on the left and right wrist. Place the heart sound sensor at the second rib level of the right edge of the sternum or the third rib level in the middle of the sternum or the fourth rib level of the left edge of the sternum. Enter the examinee's information, click the start button when each waveform is stable, and the machine will automatically analyze the results. Age and sex adjusted ba-PWV values are used to define arteriosclerosis.

#### Cardio-ankle vascular index (CAVI) evaluation

CAVI is examined by vascular equipment (VS-1500, Fukuda, Japan). Cuff, heart sound sensor and ECG electrode are placed in the same way as  ba-PWV. After observing that the waveform on the screen is stable, press the start key to start the measurement. After hearing the deflation sound, there should be six (+) signs on the screen, two in each row. Some of the examined blood vessels have severe hardening or stenosis, and there should be at least three (+) signs, one in each row. Confirm that the CAVI self-test result is "+" or "+". If it is "−" or "−", please reconfirm that the ECG electrodes, cuffs and heart sound sensors are connected correctly. CAVI > 9 on either side will be defined as arteriosclerosis.

#### Ankle–branchial index (ABI)

When detecting CAVI and ba-PWV, the value of ABI will be detected respectively. In this study, we will analyze the differences between the two kinds of ABI.

#### Carotid ultrasound detection

Carotid intima-media thickness (CIMT) and plaque are assessed with ultrasound (VIVID E80, GE, USA) with a connected electrocardiogram. The whole extracranial carotid artery was scanned by longitudinal and cross-sectional two-dimensional (2D) B-mode image, including common carotid artery, bifurcation of common carotid artery (CCA), extracranial segment of internal carotid artery (ICA) and extracranial segment of extracranial carotid artery (ECA) according to the Mannheim Carotid Intima-Media Thickness and Plaque Consensus [13].

CIMT is a double-line pattern visualized by echography on both walls of the CCA in a longitudinal image. Two parallel lines, which consist of the leading edges of two anatomical boundaries, form it: the lumen-intima and media-adventitia interfaces [13]. Edge detection system is used for semi-automatic measurements performed on a 10-mm segment of CCA instantaneously. Using the semi-automatic measurement software, the sampling frame was sampled at 1 cm in two-dimensional mode and measured the posterior wall at the bifurcation of the common carotid artery and 1 cm from the bifurcation of the common carotid artery. Measurement of CIMT should occur within a region free of plaque with a clearly identified double-line pattern in diastole on the selected frame. The measurement parameters include the average value of CIMT, the maximum value of CIMT, the minimum value of CIMT, the standard deviation of CIMT and the number of successful CIMT measurements. CIMT ≥ 1.0 mm is defined as thickening.

Plaques are focal structures encroaching into the arterial lumen of at least 0.5 mm or 50% of the surrounding CIMT value or demonstrates a thickness > 1.5 mm as measured from the intima-lumen interface to the media-adventitia interface from 2 different angles, in longitudinal and cross-sectional views [13].

The evaluation of carotid stenosis will be combined with the results of vascular diameter and area measurement and hemodynamic parameters. A peak systolic velocity (PSV) of less than 125 cm/s corresponds to lower than 50% stenosis; of 125–230 cm/s corresponds to 50–69% stenosis; and more than 230 cm/s corresponds to greater than 70% stenosis [14, 15]. The intravascular diameter method was used to evaluate the carotid stenosis using the European Carotid Surgery Trial (ECST), that is, the ratio of the residual diameter of the stenosis to the original diameter of the stenosis. The area method evaluates the stenosis rate as the area stenosis rate = [1 − (minimum lumen cross-sectional area/original lumen cross-sectional area)] × 100%.

### Beijing Vascular Health Stratification (BVHS) (see Fig. 2)

**Fig. 2:**
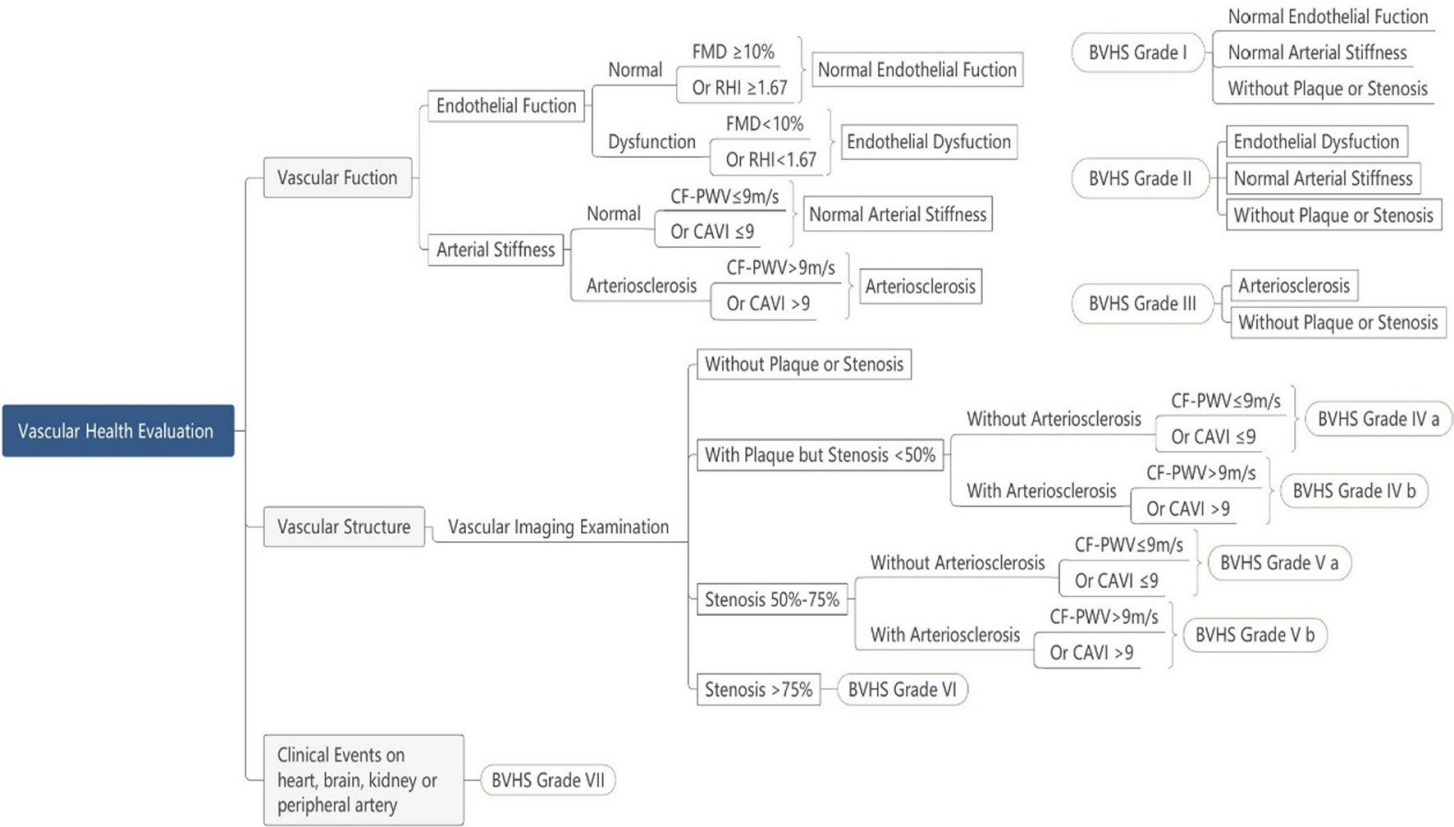
Beijing vascular health stratification (BVHS). FMD, Flow mediated vasodilation; RHI, Reactive hyperemia index; CF-PWV, Carotid-femoral artery pulse wave velocity; CAVI, Cardio-ankle vascular index

BVHS is a system to evaluate vascular health by a serious of vascular indicators, including endothelial function, arterial stiffness, atherosclerotic plaque and vascular stenosis. The standard of BVHS is showed in Fig. 2.

### End points and assessment

The primary outcome will be the incidence of MACEs.

MACEs include all cause death, cardiovascular death, non-cardiovascular death, undetermined cause of death, myocardial infarction (MI), hospitalization for unstable angina (UA), transient ischemic attack (TIA) and stroke, heart failure (HF) event, percutaneous coronary intervention (PCI), coronary artery bypass grafting (CABG), peripheral vascular intervention (PVI), stent thrombosis [16].

Cardiovascular death includes acute MI, sudden cardiac death, heart failure, stroke, cardiovascular procedure and cardiovascular hemorrhage etc.

Definition of MI is clinical syndrome where there is evidence of myocardial necrosis in a clinical setting consistent with acute myocardial ischemia, including presence of acute symptoms of myocardial ischemia, such as chest, upper extremity, mandibular, or epigastric discomfort, or an ischemic equivalent such as dyspnea or fatigue; presence of new or presumed new significant ST-segment-T wave (ST-T) changes or new left bundle-branch block (LBBB) consistent with acute myocardial ischemia; presence of new or presumed new pathological Q waves consistent with MI; presence of thrombus in a major epicardial vessel consistent with an acute MI; demonstration of a new change in myocardial viability or function consistent with MI; occurrence of an adverse angiographic finding during PCI consistent with acute myocardial ischemia; angiographic documentation of a new CABG or new native coronary artery occlusion within 48 h of CABG surgery; cardiac biomarker level [16].

Definition of hospitalization for UA include unscheduled hospitalization for the management of UA, occurring within 24 h of the most recent symptoms. Hospitalization is defined as an admission to an inpatient unit or a visit to an emergency department (ED) that results in at least a 24-h stay (or a change in calendar date if the hospital admission or discharge times are not available) [16].

Stroke is defined as an acute episode of focal or global neurological dysfunction caused by brain, spinal cord, or retinal vascular injury as a result of hemorrhage or infarction. Categorical description of stroke type classified into 1 of 3 mutually exclusive categories (ischemic, hemorrhagic, undetermined) [16].

TIA is defined as transient episode of focal caused by brain, spinal cord, or retinal ischemia without acute infarction [16].

HF event is defined as presentation of the patient for an urgent, unscheduled clinic/office/ED visit or hospital admission, with a primary diagnosis of HF, where the patient exhibits new or worsening symptoms of HF on presentation, has objective evidence of new or worsening HF, and receives initiation or intensification of treatment specifically for HF [16].

PCI is the placement of an angioplasty guidewire, balloon, or other device (e.g. stent, atherectomy, brachytherapy, or thrombectomy catheter) into a native coronary artery or CABG for the purpose of mechanical coronary revascularization [16].

CABG surgery is a procedure performed to bypass partially or completely occluded coronary arteries with veins and/or arteries harvested from elsewhere in the body, thereby improving the blood supply to the coronary circulation supplying the myocardium (heart muscle) [16].

A PVI is a catheter-based or open surgical procedure designed to improve arterial or venous blood flow or otherwise modify or revise vascular conduits. Procedures may include, but are not limited to, percutaneous transluminal balloon angioplasty, stent placement, thrombectomy, embolectomy, atherectomy, dissection repair, aneurysm exclusion, treatment of dialysis conduits, placement of various devices, intravascular thrombolysis or other pharmacotherapies, and open surgical bypass or revision [16].

### Follow up

MACEs of participants will be followed up at 6 months, 12 months, 18 months and 24 months by reviewing medical document or death certificate or by telephone visit or by clinic visit. Ba-PWV will be re-tested at 12 months and 24 months (Table 1). An adjudication committee will review all events and confirm final  diagnosis.

**Table 1:** Follow up schedule

Schedule	Baseline (July–December 2020)	Follow up			
Follow up schedule		1st follow up (June 2021)	2nd follow up (December 2021)	3rd follow up (June 2022)	4th follow up (December 2022)
Informed consent form	√				
Confirmation of inclusion and exclusion criteria	√				
Questionnaire investigation	√				
Physical examination	√				
Laboratory examination	√				
Vascular health assessment					
Endothelial function	√				
Ba-PWV	√		√		√
CAVI	√		√		√
ABI	√		√		√
Carotid ultrasound	√				
MACEs		√	√	√	√
Reasons for withdrawal or loss of follow-up		√	√	√	√

Ba-PWV, brachial ankle artery pulse wave velocity; CAVI, cardio ankle vascular index; ABI, ankle brachial index; MACEs, major adverse cardiovascular events

### Sample size estimation

About 1500 subjects will be included in this study, and the sample size was determined as follows:

### Incidence of end point events

The endpoint events concerned in this study are MACEs, the most important of which are coronary heart disease, stroke and related death. In previous studies, the standardized annual incidence of coronary heart disease in people over 45 years old in Beijing was estimated to be 497/100,000 [17], and the standardized annual incidence of stroke in people over 40 years old in China was estimated to be 533/100,000 [18]. Regardless of the difference in age composition of the population, during the 3-year follow-up period of this study, the incidence of CV events was estimated to be about 30/1000 people, and a total of about 45 cases of CV events could be obtained.

### Sample size calculation

This study is a prospective cohort study, and the main exposure factor is BVHS. BVHS is an ordered classification variable, taking the median of BVHS as the  reference, we regard the higher BVHS as the exposure group and the lower as the control group. The sample size of the cohort study was calculated by using the sample size formula of the cohort study when the sample size of the exposure group was equal to that of the control group: ${\text{n}} = 2 \times \frac{{\left( {z_{\alpha } \sqrt {2\overline{pq} } + z_{1 - \beta } \sqrt {p_{0} q_{0} + p_{1} q_{1} } } \right)^{2} }}{{(p_{1} - p_{0} )^{2} }}$, α indicates the significant level, set to 0.05; 1-β indicates the test efficacy, set to 0.9; p_0_ indicates the incidence of the control group; $q_{0} = 1 - p_{0}$; p_1_ indicates the incidence for the exposure group, $q_{1} = 1 - p_{1}$; $\overline{p} = (p_{0} + p_{1} )/2$, $\overline{q} = (q_{0} + q_{1} )/2$; The incidence of natural population events was taken as the estimation of the incidence of the control group, that is $p_{0} = 0.03$, and the risk ratio (RR) value of the exposure group was used to estimate the incidence of the exposure group. According to the previous research results of our group [10], the RR value was estimated to be 1.94, $p_{1} = p_{0} *RR$. Under the above parameters, the minimum sample size is 1471.

### Statistical analysis methods

Measurement data are expressed as mean and standard deviation (SD); classification and grade data are expressed as rate and constituent ratio. Chi-square test is used for classification and grade data; t-test or analysis of variance is used for measurement data; Pearson correlation and Spearmen rank correlation (rank data) are used for correlation analysis. For MACEs incidence during follow-up, we will use multivariate Cox regression models to test the difference between BVHS groups, adjusting for possible imbalanced variables at the baseline. We will also test if the intervention effect is modified by age, gender, cardiovascular risk factors and number of medications. Discrimination was evaluated by the C statistic. Calibration was assessed using a calibration plot. A *P* < 0.05 (two-side) is statistically significant. Data inventory and management using SAS (version 3.5.1, Vienna, Austria); all statistical analysis is completed by SAS (except for the construction of multi-level model needs to be combined with professional software MLwiN2.10).

## Results

BVHS is a potential and scientific vascular health evaluation system. The study will be the first to grade vascular health by combing various vascular indicators and explore the prediction value and compare with other risk prediction system in general Chinese population.

The baseline characteristics of study participants were showed in Table 2. From May 2020 to October 2020, a total of 1648 participants were enrolled in the study. Mean age was 60.27 ± 6.71 years, 33.9% were male. Hypertension was found in 39.3% of participants. About 19.3% were diabetes, while dyslipidemia was observed in 41.7% and 37.4% were on medication of statins. Smoking was reported by 27.1% of participants.

**Table 2:** Baseline characteristics of study participants

Variables	N	%
Age group		
< 65 years	1190	72.2
≥ 65 years	458	27.8
Sex, male	558	33.9
Hypertension	647	39.3
Diabetes mellitus	318	19.3
Dyslipidemia	687	41.7
Use of statins	615	37.4
Smoker	447	27.1

## Discussion

A unifying concept in vascular health, prolonged or chronic stimulation results in pathological remodeling and leads to formation of morphologically and functionally abnormal vessels. Healthy vessel is stable, that is the endothelium rests on a basement membrane comprised mainly of laminins and type IV collagen with associated glycoproteins but low levels of provisional matrix proteins such as fibronectin and fibrin [19, 20]. Many indicators are used to evaluate vascular stability, that is, vascular health, including endothelial function, arterial stiffness, and carotid atherosclerosis.

Studies have shown that endothelial dysfunction is associated with a number of other vascular disease and can predict CVD events [2, 21]. Recently measuring endothelial function using PAT has gained increasing attention and a proprietary device has been developed to measure observer independent pulsatile arterial volume changes by finger plethysmography (EndoPAT, Itamar Medical) [2, 22]. Andreas J et al. found non-invasive endothelial function measurements provide valuable additional information, however, to ascertain its use for daily clinical practice, future research should determine whether endothelial function can be used to guide treatment in the individual and if this translates into better outcomes [2].

Arterial stiffness is associated with cerebral small vessel disease and decreased cognitive function [23]. A meta-analysis of 17 longitudinal studies that evaluated aortic pulse wave velocity (PWV) and followed up 15,877 subjects for a mean of 7.7 years found aortic stiffness expressed as aortic PWV was a strong predictor of future CV events and all-cause mortality. The predictive ability of arterial stiffness is higher in subjects with a higher baseline CV risk [4].

A substantial global burden of carotid atherosclerosis exists. In people aged 30–79 years in 2020, the global prevalence of increased CIMT is estimated to be 27.6% (95% CI 16.9–41.3); of carotid plaque is estimated to be 21.1% (13.2–31.5); and carotid stenosis is estimated to be 1.5% (1.1–2.1) [24]. Carotid plaque and carotid stenosis are easily detected with duplex ultrasound because of the superficially positioned carotid artery. CIMT and plaque consensus suggested a standard for carotid image acquisition [13, 14]. People with carotid atherosclerosis are classified by the European Society of Cardiology as having a very high risk of death from CVD [25]. Advanced carotid atherosclerosis, defined as 50% or more stenosis, increases risk of CVD and carotid lesion-derived stroke [26].

CVD risk-assessment tools and appropriate recommendations for risk assessment in clinical guidelines are essential for implementation of a high-risk CVD prevention strategy in a population [12, 27]. Most guidelines and risk assessment models are focused on traditional CV risks, such as age, sex, SBP or hypertension, TC or ratio of TC to HDL cholesterol, smoking, HDL cholesterol, obesity, diabetes mellitus and family history of premature CVD or coronary heart disease (CHD) [27]. Although several well-known models and algorithms for CVD risk assessment have been developed [11, 27, 28], these models and guideline recommendations might not be suitable for direct application in clinical practice. In addition, an analysis of 5 risk scores, 4, including the American Heart Association (AHA) and American College of Cardiology (ACC) atherosclerotic cardiovascular disease (ASCVD) risk score, showed overestimation of risk (25% to 115%) in a modern, multiethnic cohort without baseline clinical ASCVD. If validated, overestimation of ASCVD risk may have substantial implications for individual patients and the health care system [29]. CVD risk assessment depends not only on risk-factor profile, but also on the direct vascular health risk levels, and relative risk of each vascular health factor.

The BVHS is a vascular health grading system which completely takes blood vessels as the evaluation target and takes the traditional CV risk factors as confounding factors. By collecting vascular health data for hierarchical management, a disease risk prediction model was further established. The risk of the population was divided according to the vascular health results, and different vascular health management suggestions were provided for different populations. In addition, we will provide health management follow-up platform for high-risk population, provide health science education about disease progression for high-risk population, and improve the quality of life and prevention awareness of adverse events within a controllable range. Through the construction of medical information, the information technology is applied to the management of CVD chronic diseases, and the remote management mode of CVD is constructed. This model is mainly a family-based application, which is characterized by taking the population as the basis, taking the bio-psycho-social medical model as the starting point, and taking the elimination of risk factors as the primary task of management. At the same time, we should pay attention to the relationship between clinical data and daily data, comprehensively evaluate the health problems of patients, and provide health management services for patients with CVD. Risk assessment using vascular health as an alternative endpoint index is helpful to early warning of CVD and make high-risk groups more compliant with lifestyle intervention and drug therapy. Long-term and lifelong vascular health assessment will become an important field of CV risk assessment in the future and will enter a new period of individual accurate risk assessment. Early non-invasive detection of subclinical vascular lesions is the key step to reduce the death and disability of CVD. The evaluation of vascular structure and function damage is of great value for risk stratification and curative effect judgment of patients.

## Conclusions

BVHS is a potential and scientific vascular health evaluation system. The study will be the first to grade vascular health by combing various vascular indicators and explore the prediction value and compare with other risk prediction system in general Chinese population.

## Data Availability

The datasets analyzed during the current study are available from the corresponding author on reasonable request.

## Abbreviations

BVHS: Beijing Vascular Health Stratification
CVD: Cardiovascular disease
CV: Cardiovascular
Ba-PWV: Brachial ankle artery pulse wave velocity
CF-PWV: Carotid-femoral artery pulse wave velocity
CAVI: Cardio ankle vascular index
ABI: Ankle brachial index
FMD: Flow mediated vasodilation
RHI: Reactive hyperemia index
IMT: Intima-media thickness
MACEs: Major adverse cardiovascular events
eGFR: Estimated glomerular filtration rate
CRF: Case record form
EDC: Electronic data capture
SBP: Systolic blood pressure
DBP: Diastolic blood pressure
TC: Total cholesterol
TG: Triglyceride
LDL-C: Low density lipoprotein cholesterol
HDL-C: High density lipoprotein cholesterol
CRP: C-reactive protein
HCY: Homocysteine
FPG: Fasting plasma glucose
ALT: Alanine aminotransferase
AST: Aspartate aminotransferase
HbA1c: Glycosylated hemoglobin
PAT: Peripheral arterial tonometry
ECG: Electrocardiogram
CIMT: Carotid intima-media thickness
CCA: Common carotid artery
ICA: Internal carotid artery
ECA: Extracranial carotid artery
PSV: Peak systolic velocity
MI: Myocardial infarction
UA: Unstable angina
TIA: Transient ischemic attack
HF: Heart failure
PCI: Percutaneous coronary intervention
CABG: Coronary artery bypass grafting
PVI: Peripheral vascular intervention
CHD: Coronary heart disease
ASCVD: Atherosclerotic cardiovascular disease
